# Gender differences in brain activity underlying acupuncture sensations at LR3: a task-based fMRI study

**DOI:** 10.3389/fnhum.2025.1649644

**Published:** 2025-09-09

**Authors:** Hao Chu, Bin-Jian Jiang, Dong-Na Li, Wen-Xiu Duan, Zi-Zhan Gao, Yan-Yan Yang, Ning Dai, Chun-Sheng Xu, Zi-Jian Wu

**Affiliations:** ^1^School of Acupuncture and Tuina, Anhui University of Chinese Medicine, Hefei, China; ^2^Anhui Province Key Laboratory of Meridian Viscera Correlationship, Hefei, China; ^3^Department of Acupuncture and Moxibustion, Gansu Province Hospital of Traditional Chinese Medicine, Lanzhou, China; ^4^College of Traditional Chinese Medicine, Nanjing University of Chinese Medicine, Nanjing, China; ^5^Department of Radiology, The First Affiliated Hospital of Anhui University of Chinese Medicine, Hefei, China; ^6^Department of Andrology, The First Affiliated Hospital of Anhui University of Traditional Chinese Medicine, Hefei, China

**Keywords:** acupuncture, gender difference, task-based magnetic resonance imaging, functional connectivity, fMRI

## Abstract

**Background:**

Acupuncture has been practiced in China for centuries, and LR3 (Taichong) is a key acupoint of the liver meridian that has been widely studied. Whether its central mechanism differs by sex remains unclear, because earlier work used single-sex or mixed-sex samples without stratified analysis. To close this gap, we used task-based fMRI together with the Massachusetts Acupuncture Sensation Scale to compare sex differences in brain activation and thalamic functional connectivity.

**Methods:**

We enrolled 30 men and 25 women in a block-design acupuncture task, the modular task-based functional magnetic resonance imaging (fMRI) technique was employed to investigate brain activity, and the Massachusetts Acupuncture Sensation Scale (MASS) was utilized to assess acupuncture sensations experienced by the participants. Alterations in activated brain regions and functional connectivity were examined separately for males and females. Statistical analyses were performed to determine potential gender differences in acupuncture sensation.

**Results:**

Significant statistical differences were observed in the 12 acupuncture sensation scores of the MASS scale (*p* < 0.001). Acupuncture LR3 activated the left inferior cerebellar gyrus, right superior margin gyrus, and left posterior central gyrus in women, and activated the right superior margin gyrus, left central sulcus, and right paracentral lobule in men. Enhanced functional connectivity was also observed in the bilateral thalamus of women: the bilateral caudate nucleus, right precuneus, right cuneate fissure, and bilateral lingular gyrus were highly active. Furthermore, the right thalami also demonstrated enhanced functional connectivity with the left cuneate fissure, right posterior cingulate gyrua, and left precuneu.

**Conclusion:**

The differences in the functional connectivity of the right superior marginal gyrus and thalamus between men and women may account for the differences in acupuncture sensory processing between men and women. In addition, the right superior marginal gyrus may be a specific brain region critical for activation of the LR3 acupoint.

## 1 Introduction

Acupuncture has a long history in China and is a highly regarded traditional therapy. Research indicates that acupuncture is closely associated with the central nervous system (Yan et al., 2022). Functional magnetic resonance imaging (fMRI) is a non-invasive visualization technique developed in the 1990s that can real-time display the brain's response to external stimuli. This approach is widely used to study acupuncture's central nervous system mechanisms, including the activation of specific brain regions (Wong et al., 2023). Acupuncture enhances functional connectivity in networks such as the limbic-paralimbic-neocortical network, the default mode network (DMN), and the sensorimotor network (SMN), and increases the correlation between limbic/paralimbic regions and subcortical structures (such as the insula, amygdala, and thalamus) in healthy individuals (Fang et al., 2009). In traditional Chinese medicine theory, the liver is responsible for “regulating the flow of qi,” which plays a crucial role in regulating emotional states and responding to external stimuli. Notably, the insula and thalamus are functionally closely related, particularly in processing bodily sensations and emotional generation, whose functions highly correspond to the liver's role in “regulating emotional states.” The Tai Cong acupoint (LR3), as the original acupoint of the liver meridian, has been extensively studied due to its unique characteristics in the traditional Chinese medical theory system. In his study on the LR3, (Rao et al. 2024) proposed that acupuncture at theLR3 can significantly activate the left thalamus. (Geng et al. 2021) observed that in patients with irritable bowel syndrome with diarrhea (IBS-D), acupuncture at bilateral LR3 and Baihui acupoint (GV20) resulted in enhanced functional connectivity (FC) between the right insula and left hippocampus, and reduced FC between the left insula and right hippocampus, improving abdominal pain, bloating, and emotional state in IBS-D patients. This suggests that the LR3 has a definite regulatory role in pain processing and emotional regulation. More importantly, in the field of pain, numerous studies have clearly demonstrated that women generally exhibit higher sensitivity to various pain stimuli than men, and the neural representation of pain experiences also exhibits gender specificity (Fillingim et al., 2009). Similarly, some scholars have observed that female RA patients experience more severe and widespread pain sensitivity, a difference independent of inflammation, suggesting gender-related differences in pain regulation mechanisms (Vogel et al., 2023).

However, previous studies on the neural mechanisms of acupuncture at the LR3 have mostly used single-sex or mixed-sex samples without conducting stratified analysis, failing to adequately consider the potential influence of gender—a critical factor—on acupuncture-induced brain network remodeling (Zhang et al., 2022) particularly in networks related to pain and emotion processing. In fact, gender differences may exist in emotion processing, memory formation, perceptual abilities, and language skills. Resting-state magnetic resonance imaging studies have revealed significant differences between men and women in specific brain regions, including the inferior frontal gyrus, anterior cingulate cortex, hippocampus, central posterior gyrus, cerebellar Crus I-II, lobule VI VIIb, and posterior vermis VI-X (Brennan et al., 2021; Xin et al., 2019; Gao et al., 2022).

The thalamus-cortical projections transmit nearly all incoming information to the cerebral cortex and mediate cortico-cortical information transmission (Habig et al., 2022). The thalamus has extensive connections with multiple brain structures and may serve as a major hub for functional brain networks. The lateral thalamus (primarily comprising the ventral lateral thalamus) is the primary sensory nucleus projecting to somatosensory cortex; the medial thalamus is associated with pain and emotion and connects to the limbic cortex. As the gateway to the cerebral cortex, the thalamus serves as a critical relay station for the transmission of noxious information and regulates pain awareness. Needle-prick sensation is a complex experience resulting from the interaction of multiple sensory modalities, with pain as the primary component, playing a key role in the complex processing of needle-prick stimuli. This study hypothesizes that there are gender differences in the processing of needle-prick stimuli, and that the functional connectivity patterns of the thalamus may differ between men and women.

Prior research mainly examined resting-state brain changes before and after acupuncture, with limited attention to real-time changes during acupuncture. Therefore, this study employed task-based testing to investigate the effects of acupuncture on brain activity. (Cole et al. 2021) found that task-related functional connectivity changes play a crucial role in dynamically reshaping brain network structures and directing neural activity flows during task execution. (Bernstein-Eliav and Tavor 2024) suggested that connectivity patterns may serve as the foundation for brain activity, and task-specific brain connectivity patterns can be used to predict regional brain activity during the execution of various cognitive tasks. Therefore, this study employed functional connectivity methods to examine acupuncture's effects on brain regions and the differences of gender using neural networks.

## 2 Materials and methods

### 2.1 Subjects

A total of 30 men and 25 women were recruited from the First Affiliated Hospital of Anhui University of Traditional Chinese Medicine. Participants included in the study were selected according to the following criteria: 1. age: 20–35 years old; 2. right-handed; 3. no acupuncture 1 month before the experiment; 4. Patients had not taken any medications for 30 days prior to enrollment and during the trial period.; 5. no history of alcohol, drug dependence, or head surgery; 6. no contraindications to MRI scanning; 7. able to tolerate the noise generated by the MRI machine during testing; 8. no claustrophobia; 9. women were not menstruating; 10 signed the informed consent before experimentation. This study was approved by the Ethics Committee of the First affiliated Hospital of Anhui University of Traditional Chinese Medicine (Approval No.: 2021AH-78).

Once participants had been enrolled in the study, they were excluded from data analysis if any of the following conditions occurred during the scanning process: 1. poor compliance, which affected data collection according to the operation instructions; 2. Excessive head movement (head movement >3° or 3 mm); 3. abnormal body movement occurring during the scanning process; 4. subjects withdrew voluntarily due to personal reasons.

### 2.2 Stimuli and functional paradigm

**Figure 1:**
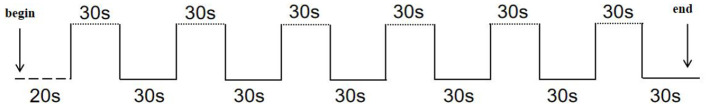
Experimental flow chart. The dashed line indicates the baseline state and the solid line indicates the task state.

All participants were scanned in the same way. The task mode was designed modularly, with 12 parts in total, which were alternated by 6 30-s “baseline” states and 6 30-s “task” states. Importantly, 20 s data were added at the beginning and were removed at the later analysis to avoid the influence of unstable data at the beginning of the scan (Figure 1).

**Figure 2:**
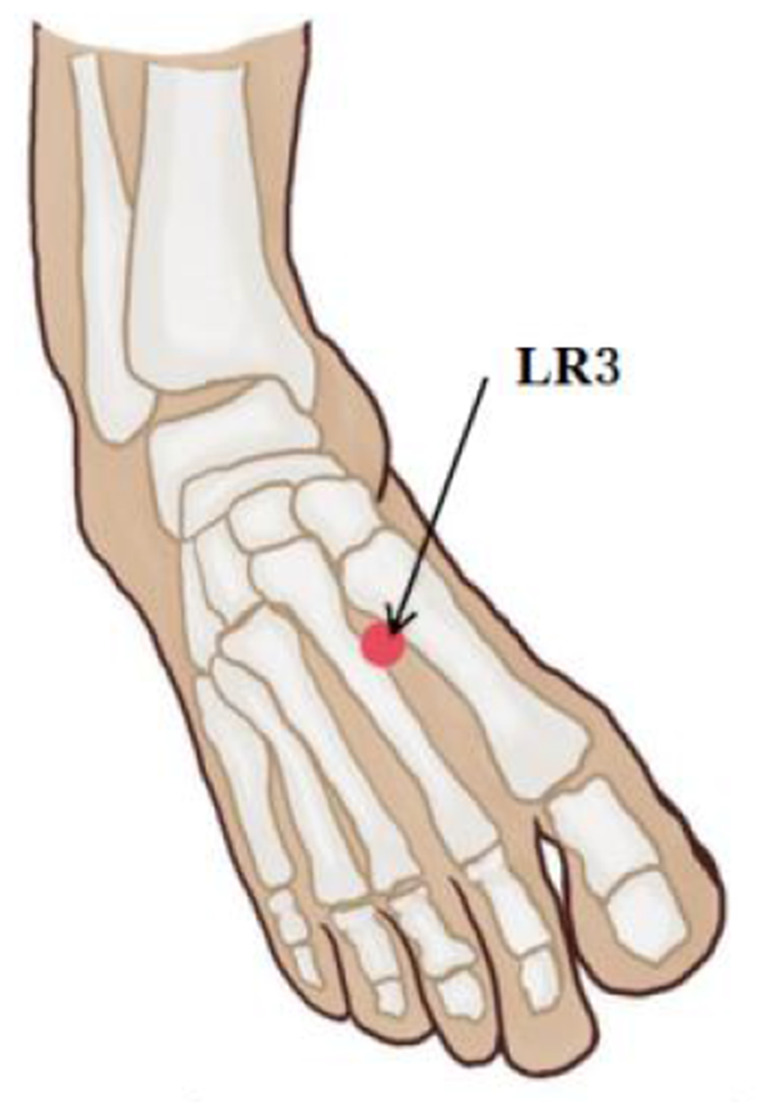
Acupuncture point selection.

The left LR3 acupoint, located in the foot, was utilized during this experiment due to its relative lack of interference with the magnetic field during fMRI scanning. LR3 was selected based on the World Health Organization (WHO) acupuncture point positioning metered, and it was positioned at the depression before the junction of the first and second metatarsal bones on the dorsal side of the left foot (Figure 2). All experimental operations were conducted by a professional acupuncturist for consistency purposes. Subjects were informed prior to scanning that different sensations may occur during acupuncture process with feelings scored from 0 to 10 using MASS scale (Kong et al., 2007) including pain, soreness, pressure, heaviness, fullness, warmth, coolness, numbness, tingling, and dull pain; an experimenter asked the participants if they experienced any of these sensations after each scan. The higher the score, the more significant was the subject's perception of the corresponding stimulus.

The brain magnetic resonance data of all subjects were collected by a GE Discovery MR750 3.0T magnetic resonance scanner in the United States. The subjects were required to relieve themselves before the experiment and wear eye masks and earplugs to reduce audiovisual interference. Their heads were placed in foam headrests to limit movement. The experimenter was stationed next to the scanning instrument and performed acupuncture controls according to the display time. Prior to scanning, strict sanitary procedures were followed for both experimenter's hands and subjects' skin around acupoints. Hua Tuo acupuncture needles (0.25 mm × 25 mm) were used with a controlled depth of insertion at 15 mm ± 2 mm straight into needle insertion point. Experimentation began once needle sensation had been produced. In “task” mode, the twisting angle was controlled at (180 ± 5)° with the frequency set at (60 ± 5) times/min, and the time was 30 s; while in “baseline” mode, no torsion was given to needles, and the duration was 30 s. This sequence was repeated 6 times, with an additional 20 s of rest at the beginning of each run, and the total scan time for each run was 380 s.

### 2.3 Data collection

T1 structural data scanning parameters were as follows: pulse repetition time (TR) = 8.2 ms, echo time (TE) = 3.2 ms, scanning matrix = 256 × 256, flipping angle (FA) = 12°, field of view (FOV) = 256 × 256 mm^2^, layer thickness 1 mm, no layer spacing, and the number of layers was 166. voxel size 1.0 mm × 1.0 mm × 1.0 mm.

Task-state data scanning parameters were as follows: pulse repetition time (TR) = 2,000 ms, echo time (TE) = 30 ms, scanning matrix = 64 × 64, flipping angle (FA) = 90°, field of view (FOV) = 220 × 22 mm^2^, layer thickness 3.0 mm, layer spacing 1 mm, resolution = 64 × 64, the number of layers was 36, voxel size 3.44 mm × 3.44 mm × 3.0 mm and the acquisition time was 380s. Data analysis is presented in Supplementary material.

### 2.4 Data pre-processing

Use SPM12 (https://www.fil.ion.ucl.ac.uk/spm/software/spm12/) based on MATLAB 2017b (Mathworks Inc., USA) to convert images (DICOM to NII), and perform image pre-processing on the converted data, including: Time layer correction: remove the first 10 time points; Head motion correction: Remove data from subjects with translation >3 mm or rotation >3°; Spatial standardization: Select the DARTEL method for registration; Apply Gaussian smoothing using a Gaussian kernel (full width at maximum = 6 mm). Based on these pre-processing steps, we further use linear regression to remove irrelevant noise time series (including head motion-related Friston-24, cerebrospinal fluid, and white matter signals, as well as their time-differenced signals (derivatives) and task-related signals.

Fixed-effects models were used to analyze individual comparisons of acupuncture, while random-effects models were employed for group data analysis. The Boxcar effect was modeled using a convolution with a typical hemodynamic response function. For FC processing, we fully referenced (Cole et al. 2019) and utilized the FIR task regression method, which has been extensively applied in task-based functional connectivity analysis. Following the regression step, the detrend function was applied to remove the linear trend from the time-series signals. Finally, considering the possibility of task-related signals at high frequencies, we applied a bandpass filter with a bandwidth of 0.2–0.008 Hz. Additionally, we selected seed points based on the AAL atlas (https://www.gin.cnrs.fr/en/tools/aal/), extracted the right supramarginal gyrus and bilateral thalamus as templates, and for each subject, extracted the average time-series signal from each brain region. We then used the Pearson correlation algorithm to calculate the functional connectivity values from each brain region to every voxel Whole-brain analysis used Fisher's Z-transformed correlation coefficients (R → Z) for statistics.

### 2.5 Statistical analysis

SPSS 26.0 was used to conduct statistical analyses. Demographic information is presented as mean ($\bar{\text{x}}$ ± s) standard deviation. Inter-group comparisons were analyzed via two-tailed independent samples *t*-tests at *p* ≤ 0.05 (two-sided test). For group analysis of imaging data, paired sample *t*-test was employed with a significance level of *p* < 0.05. After obtaining whole brain functional connectivity maps for each subject's seed point, we calculated the group average functional connectivity map using single-sample *t*-test for each group of subjects and compared inter-group differences between male and female groups at each seed point using a two-sample *t*-test. Strict multiple comparison correction adjusted the difference to *p* < 0.01. The cluster-level multiple comparison correction method was also applied to each type of brain map with cluster *p* < 0.01 and voxel *p* < 0.001 and was considered significant for inter-group differences detection.

## 3 Results

All participants completed the scan. Three males and four females were excluded due to excessive head movement, and two males were excluded due to partial image loss. A total of 25 males and 21 females were included in the final analysis.

Demographic data (age: 27.25 ± 1.7 years for males, 26.2 ± 1.881 years for females; years of education: 19.42 ± 2.466 years for males, 20.1 ± 2.269 years for females) showed no statistically significant differences (*p* > 0.05).

### 3.1 Needle sensation score statistics

In the 12 acupuncture sensation scores, there were significant differences between males and females in soreness, pain, sharp pain, tingling, numbness, pressure, heaviness and swelling (*p* < 0.001). Under the same stimulation, females felt more soreness, pain, sharp pain, tingling and numbness, while males felt more pressure, heaviness and swelling (Table 1).

**Table 1:** MASS acupuncture sensation scale statistics.

**Acupuncture sensation**	**Male (*n* = 25)**	**Female (*n* = 21)**	***P* size**
Aching	3.96 ± 1.485	5.81 ± 1.47	< 0.001
Deep pressure	4.24 ± 1.715	2.143 ± 1.824	< 0.001
Heaviness	4.52 ± 1.896	2.667 ± 1.065	< 0.001
Fullness	4.88 ± 1.833	5.238 ± 1.411	< 0.001
Warmth	2.56 ± 1.781	2.143 ± 1.558	0.4071
Cold	2.36 ± 2.119	2.048 ± 1.499	0.574
Numbness	2.4 ± 1.472	4.905 ± 1.895	< 0.001
Tingling	2.44 ± 1.805	4.81 ± 1.601	< 0.001
Dull pain	2.76 ± 1.562	3.429 ± 1.989	0.2084
Soreness	1.44 ± 1.158	3.381 ± 1.884	< 0.001
Sharp pain	1.84 ± 1.248	4 ± 1.643	< 0.001
Throbbing	2.4 ± 1.414	2.048 ± 1.499	0.4172

### 3.2 Brain activation area

#### 3.2.1 The female group activated regional results

**Figure 3:**
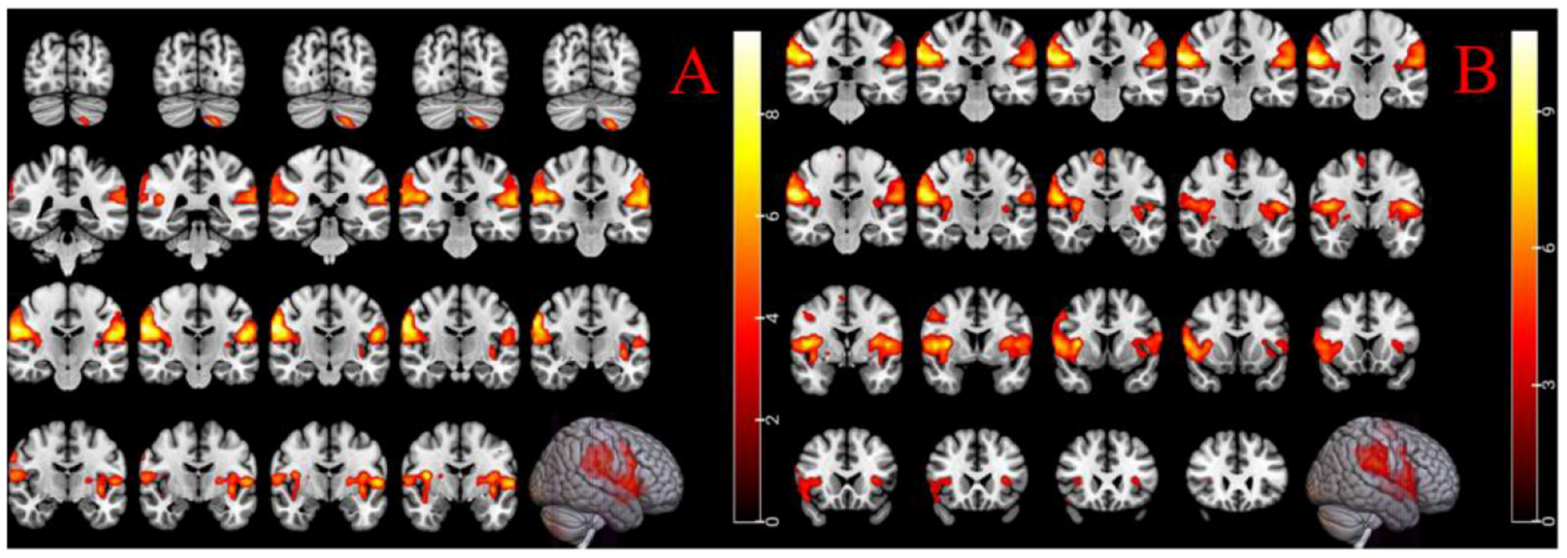
**(A)** Acupuncture in female LR3 activated brain region; **(B)** acupuncture activates the brain area of male LR3. The right color band indicates the degree of activation, voxel level *p* < 0.001, cluster level *p* < 0.05 (FWE correction).

Female-specific brain regions activated by LR3 acupuncture were left inferior cerebellum, right supramarginal gyrus, and left central posterior gyrus (Table 2, Figure 3).

**Table 2:** Brain function activation area of LR3 in female and male acupuncture groups.

**Gender**	**Region label**	***F*-value**	**Cluster size**	**MNI**		
				***X***	***Y***	***Z***
Female	Cerebelum_8_L	6.2024	23	−18	−78	−51
	SupraMarginal_R	9.6608	292	66	−15	27
	Postcentral_L	8.6439	88	−54	−18	18
Male	SupraMarginal_R	10.8782	368	69	−24	27
	Rolandic_Oper_L	8.1561	158	−48	0	6
	Paracentral_Lobule_R	6.9088	89	9	−15	63

#### 3.2.2 Male group activation area results

Male-specific brain regions activated by LR3 acupuncture were the right supramarginal gyrus, the left central tegmental sulcus, and the right paracentral lobule (Table 2, Figure 3).

### 3.3 Function binding

#### 3.3.1 Differences between groups in FC of the right supramarginal gyrus

**Figure 4:**
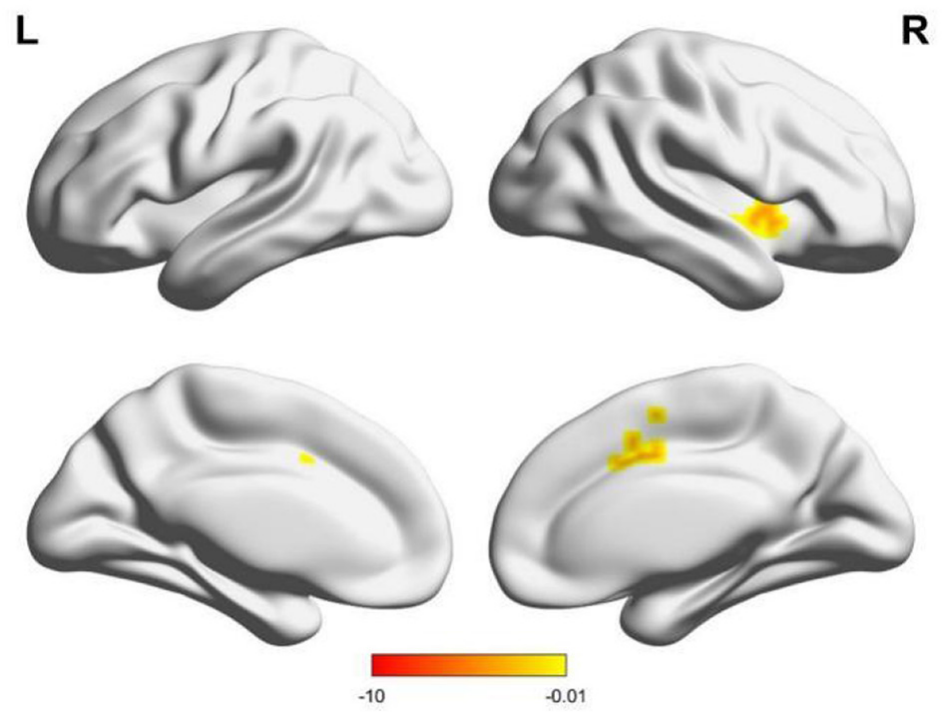
Changes in brain functional connections with the right supramarginal gyrus as seed points, and the lower color band represents the *T*-value.

Compared to males, females had enhanced functional connectivity between the right supramarginal gyrus and the left cerebellum, the right middle cingulate gyrus, and the right putamen (Table 3, Figure 4).

**Table 3:** Functional connectivity differences in the right superior limbic gyrus between men and women.

**ROI**	**Region label**	**Extent**	***t*-value**	**MNI**		
				***x***	***y***	***z***
SupraMarginal_R	Cerebellum_6_L	36	5.500	−24	−60	−30
	Cingulate_Mid_R	44	4.882	9	6	33
	Putamen R	41	4.864	39	−12	−9

#### 3.3.2 Bilateral thalamic FC differences between groups

**Figure 5:**
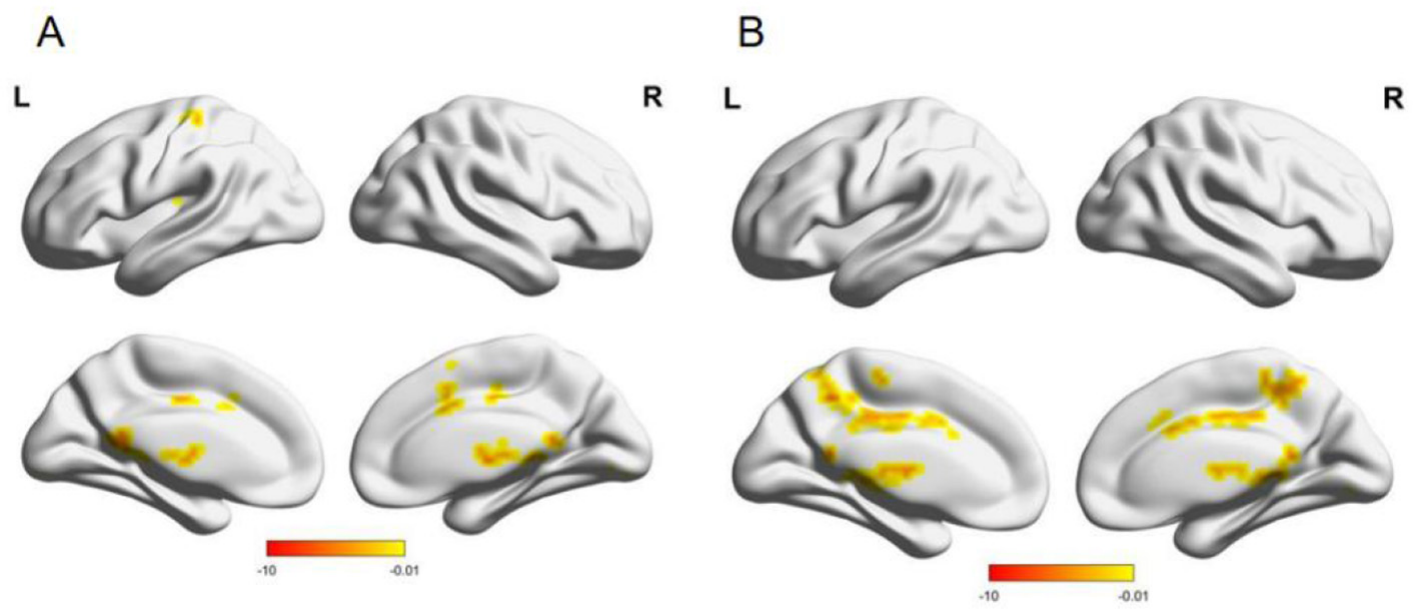
**(A)** Brain functional connection with the left thalamus as the seed point; **(B)** Brain functional connection with the right thalamus as the seed point; L, left; R, The color bar at the bottom right indicates the magnitude of the T-value.

Compared with males, females had enhanced functional connectivity between the left thalamus and bilateral caudate nucleus, right precuneus, right raphe, bilateral gyrus linguae, left posterior central gyrus, and left supplementary premotor area; the right thalamus and left raphe, right posterior cingulate gyrus, right gyrus linguae, left precuneus, and left paracentral lobule (Table 4, Figure 5).

**Table 4:** Differences in bilateral thalamic functional connections between men and women.

**ROI**	**Region label**	**Extent**	***t*-value**	**MNI**		
				***x***	***y***	***z***
Thalamus_L	Caudate_L	176	7.383	−18	9	15
	Caudate_R	176	5.739	12	3	12
	Precuneus_R	176	5.287	9	−39	3
	Calcarine_L	197	6.592	−3	−57	3
	Lingual_R	197	5.565	6	−78	−6
	Lingual_L	197	4.523	−12	−39	−3
	Postcentral_L	44	5.482	−30	−30	54
	Supp_Motor_Area_L	47	5.135	0	12	45
Thalamus_R	Calcarine_L	118	9.999	−3	−57	3
	Cingulate_Post_R	118	4.803	9	−42	21
	Lingual_R	123	7.194	9	−42	3
	Precuneus_L	342	6.735	−9	−48	45
	Paracentral_Lobule_L	342	6.093	−6	−21	54

## 4 Discussion

This study investigated gender differences in brain activity during acupuncture in men and women. In women, acupuncture at theLR3 activated the left inferior cerebellar hemisphere, right superior temporal gyrus, and left postcentral gyrus; in men, acupuncture at theLR3 activated the right superior temporal gyrus, left central sulcus, and right paracentral lobule. Although both sexes activated the right superior temporal gyrus, the activation was stronger in men. When analyzing functional connectivity patterns with the right superior temporal gyrus as the seed point, enhanced functional connectivity was observed between the right superior temporal gyrus and the left cerebellum, right middle cingulate gyrus, and right putamen in females. When using the bilateral thalamus as another seed point, it was found that the left thalamus in females showed enhanced functional connectivity with the bilateral caudate nuclei, right precuneus, and lingual gyrus, and also had connections with the left central posterior gyrus and supplementary motor area; conversely, the right thalamus in males showed enhanced functional connectivity with the left parahippocampal gyrus, posterior cingulate cortex, lingual gyrus, anterior cingulate cortex, and paracentral lobule.

This finding is not merely an anatomical discovery, but is highly consistent with traditional Chinese medicine theory. The liver meridian “rises from the forehead and meets the governor vessel at the vertex,” while the right superior temporal gyrus is located at the junction of the parietal, temporal, and occipital lobes, which is precisely the core region responsible for “integrating visual, auditory, and sensory information, maintaining bodily boundaries, and harmonizing the self with others.” Wada's research also indicates that the volume of the right superior temporal gyrus is closely related to emotional recognition ability (Wada et al., 2021). The female-specific activation of the left inferior cerebellar hemisphere, right superior temporal gyrus, and left posterior central gyrus corresponds to the functions of the liver meridian in “regulating blood, regulating the spirit, and regulating bodily sensations,” and can be regarded as the brain's reflection of the liver's role in “regulating the flow of energy.” The Suwen: On the True Nature of the Ancient Times states that “women are born with the liver,” suggesting that the physiological and pathological functions of women are closely related to the liver. The liver stores blood, regulates the flow of qi, and harmonizes the Chong and Ren vessels, hence acupuncture at the LR3 is particularly effective for women in “regulating the blood sea and soothing emotions.” In contrast, the enhanced connections between the right thalamus and the wedge lobe, posterior cingulate gyrus, anterior wedge lobe, and paracentral lobule in males highlight the functional integration of the “thalamus-parietal lobe-motor cortex” pathway, manifesting as the “liver qi regulation leads to smooth movement of tendons and vessels” effect on movement and the body.

It is worth noting that variations in needle sensation occurred during acupuncture, a finding we also observed in our experiment. While the MASS scale effectively assessed needle sensation in both sexes (indicating generally similar subjective experiences), we identified gender differences in specific sensory components related to acupuncture pain. Specifically, women reported significantly greater intensity for sensations like sharp pain, sourness, numbness, and distension compared to men. This finding closely aligns with the gender differences reported by (Yeo et al. 2016a) at the Yanglingquan acupoint (SP9), and is supported by extensive research in pain science: Bartley and Fillingim's systematic review found that women generally exhibit lower pain thresholds and tolerance (Bartley and Fillingim, 2013). (Fillingim et al. 2009) further emphasized that cognitive-emotional factors (such as fear, anxiety, and disgust) are key drivers of gender differences in the central processing of pain. Pain, as a subjective and multidimensional experience encompassing sensory discrimination, emotional motivation, and cognitive evaluation dimensions (Reddan and Wager, 2018), exhibits cross-gender commonalities in its neural processing mechanisms such as transduction, transmission, regulation, and perception. However, the involvement and mechanisms of emotional states (Gilam et al., 2020) and cognitive regulation may exhibit gender-specific differences, which may constitute the core mechanisms underlying women's higher reported pain sensitivity.

Neuroimaging results provide a more reasonable neurobiological explanation for the gender differences we observed. When acupuncture was applied to theLR3, we observed distinct brain activation patterns: females exhibited specific activation of the central posterior gyrus (primary sensory cortex, SI), while males primarily activated the paracentral lobule. The central posterior gyrus is a key region for somatosensory processing, with its activity intensity positively correlated with subjective pain perception levels and involvement in encoding pain-related sensory features such as intensity, localization, and regulating non-painful tactile perceptions (Sun et al., 2023; Peyron et al., 2000; Kropf et al., 2019). The significant activation of this region in females may reflect a broader range of pain perception and a stronger tendency toward cognitive evaluation of pain stimuli, i.e., a greater focus on the overall intensity and emotional significance of pain. The paracentral lobule plays a key role in sensation and movement of the lower limbs. Many studies have shown that the paracentral lobule is activated when acupoints on the lower limbs and feet are stimulated (Yeo et al., 2016b; Yan et al., 2005; Ren et al., 2012). The paracentral lobule is primarily responsible for precise sensory-motor integration in the lower limb region. Its dominant activation in males may indicate that males have more precise spatial localization abilities for pain stimuli and place greater emphasis on the spatial attributes of sensory information.

We observed significant activation of the right superior temporal gyrus in both the male and female groups, highlighting the importance of this region as a key response target for acupuncture at theLR3. In-depth functional connectivity analysis revealed gender-specific network interaction patterns: in the female group, the right superior temporal gyrus showed significantly enhanced connectivity with the left cerebellum, right cingulate gyrus (particularly the anterior-middle cingulate gyrus), and right putamen. The cingulate gyrus (especially the middle cingulate gyrus) plays a central role in the affective motivational components of pain and in the cognitive assessment and response to threatening stimuli such as pain (Oane et al., 2023; Rolls, 2019). The cerebellum is involved in sensory prediction and emotional regulation, while the putamen belongs to the basal ganglia circuit. Therefore, the enhanced connectivity pattern observed in the female group strongly suggests that the cognitive-emotional network is crucial in the acupuncture pain experience of women.

The thalamus, as a key relay station and integration center for sensory information transmission to the cortex, demonstrated enhanced functional connectivity with multiple higher-order functional networks (including the salience network, default mode network, and cortico-striatal loop) in this study. This is highly consistent with its central role in pain signal processing: the thalamus not only receives and transmits nociceptive information (Qin et al., 2020), but also participates in pain perception, regulation, and the integration of pain with other cognitive processes (such as attention and emotion) (Habig et al., 2022; Tracey, 2010). For example, (Habig et al. 2022) observed bilateral activation of the thalamus under mechanical pain stimuli, while (Tracey 2010) found widespread activation of the thalamus during various pain experiences (including imagined pain) and their cognitive regulation. The enhanced thalamic network connectivity observed in this study further supports its role as a hub in coordinating pain-related cognitive and emotional regulation. Additionally, the involvement of the supplementary motor cortex and caudate nucleus, as key nodes in the “cortico-basal ganglia-thalamic-cortical” loop, suggests that acupuncture responses also involve the integration of motor preparation, autonomic nervous responses, and higher cognitive functions.

Previous fMRI studies on the LR3 (acupoint, Kong et al., 2023; Zheng et al., 2016; Qiu et al., 2012) reported activation or functional connectivity changes involving regions such as the thalamus, frontal lobe, somatosensory cortex, cingulate gyrus, temporal lobe, and parietal lobe. However, most of these studies did not explicitly distinguish between genders or used mixed samples. (Qiu et al. 2010) directly compared gender and found that under acupuncture and moxibustion combined intervention, men and women exhibited differences in activation intensity in the lateral prefrontal network (LPNN) and default mode network (DMN). (Li et al. 2018) studied acupoints on the lower limb knee joint and found that males exhibited stronger activation in the right middle frontal gyrus, inferior frontal gyrus, precuneus, superior parietal lobule, and anterior cerebellar lobe, while females showed stronger activation in the right frontal lobe, parietal lobe, and middle temporal gyrus, but deactivation in the medial frontal lobe. These independent studies have confirmed the gender-specific patterns observed in this study in key brain regions such as the frontal lobe, parietal lobe, and cingulate gyrus, collectively explaining the existence of neurobiological gender differences in acupuncture responses. In fact, gender differences in brain responses have been reported multiple times in studies investigating acupuncture treatment for insomnia, depression, and anxiety disorders (Bartley and Fillingim, 2013; Fan et al., 2015), further emphasizing the necessity of considering gender factors when understanding and optimizing acupuncture's therapeutic effects.

## 5 Conclusion

By integrating the analysis of the MASS scale and neuroimaging results, the core findings of this study are as follows: women exhibited higher pain sensitivity but relatively weaker spatial localization accuracy during acupuncture, while men demonstrated stronger pain stimulus localization ability but lower subjective intensity reports. These differences primarily stem from fundamental gender-based disparities in the brain's perception, evaluation, and integration of pain information. The right superior temporal gyrus, as a common activation site, and its gender-specific connectivity patterns may serve as important neural nodes mediating these differences. Of course, we do not rule out the influence of other factors. Xu's research suggests that higher circulating testosterone levels in men may increase their pain threshold by enhancing opioid system-mediated analgesic effects (Xu et al., 2024). (Lund and Lundeberg 2008) further notes that women may be more prone to adopting a “catastrophizing” cognitive pattern in painful situations, amplifying pain perceptions. Additionally, societal and cultural norms that expect men to be “strong and tolerant of pain” may suppress their pain reporting behavior. These neurobiological, cognitive psychological, and sociocultural factors may collectively shape the observed gender differences.

This study provides preliminary evidence for understanding the neural mechanisms underlying gender differences in acupuncture. However, several limitations exist, primarily including: a relatively small sample size, necessitating future studies with larger samples to enhance the robustness and generalizability of the results; and a narrow focus on the LR3 to discuss the physiological functions of the liver meridian, as the liver meridian's physiological functions constitute a complex network mediated by multiple acupoints along its course. Different acupoints, due to their specific attributes and primary therapeutic characteristics, may exhibit distinct stimulation effects and potential gender-specific patterns. Future studies should aim to optimize these aspects and explore the potential of gender-specific neural response patterns as biomarkers for predicting acupuncture efficacy, thereby providing comprehensive and objective neuroscientific evidence for the development of personalized and precise acupuncture treatment protocols. In future acupuncture basic research, clinical trials, and clinical practice, the gender perspective should be fully considered and incorporated.

## Data Availability

The raw data supporting the conclusions of this article will be made available by the authors, without undue reservation.
