# Supplementary material for: Gender differences in brain activity underlying acupuncture sensations at LR3: a task-based fMRI study
Source: Front Hum Neurosci. 2025 Sep 9;19:1649644. doi: 10.3389/fnhum.2025.1649644 (PMC12454379; doi:10.3389/fnhum.2025.1649644)
Supplement: Supplementary file 1 [file Supplementary_file_1.zip › Supplementary Material/Table_1.DOCX]

**Differential brain region analysis process**

**1. Project Basic Information**

The original data consisted of two groups of subjects: 30 males and 25 females. After removing subjects with head movements greater than 3 mm, 47 subjects remained for statistical analysis: 25 males and 21 females.

**2. Data Preprocessing**

The basic data parameters are as follows:


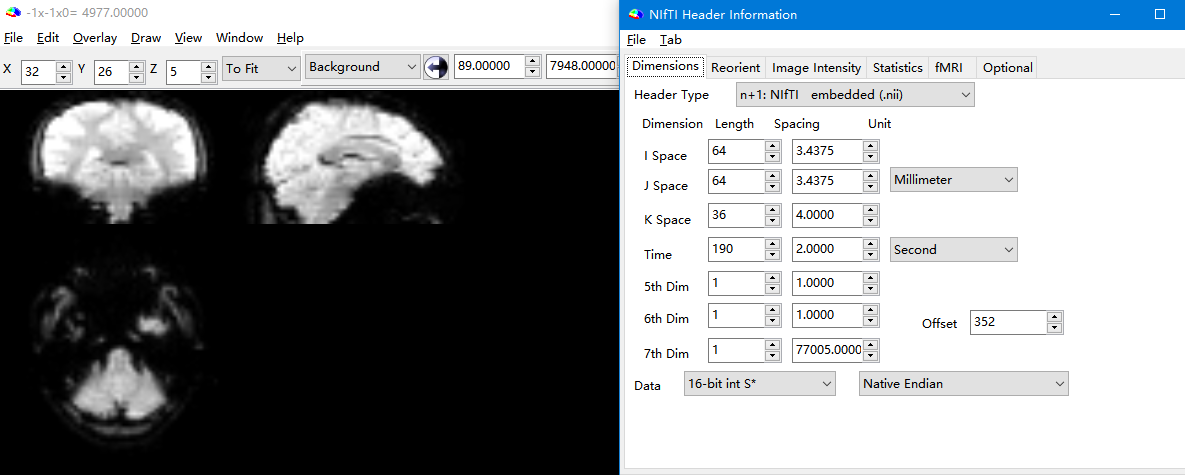


T Delete the first 10 time points because the scan has just started and the signal has not yet reached a stable state. The subject also needs some time to adapt to the scan noise. According to the experimental design, delete the first 20 seconds of the resting state.

A Slice timing Time layer correction (36 layers, layer sequence [2:2:36 1:2:35], reference 36)

When an MRI machine scans, it acquires images of the brain layer by layer, rather than obtaining a complete image of the brain instantaneously. However, post-processing software assumes that the 3D brain is acquired simultaneously, so time layer correction is required to use interpolation to make all layers appear as if they were acquired simultaneously.

R realign Head motion correction

Using the average functional image as a reference, adjust the position of the brain at each time point to ensure that the data direction remains consistent across all time points, reducing noise caused by head motion during scanning. At the same time, record the degree of head motion for each subject, which is used as a standard to assess data quality and remove subjects with excessive head motion.

W normalize Spatial normalization Select the DARTEL method for registration.

S smooth Spatial smoothing Select a smoothing kernel (FWHM) of 6*6*6 mm. This is to reduce the deformation noise introduced by the previous spatial normalization process and to make the data distribution more normal, facilitating subsequent statistics.

After data preprocessing is complete, check the head motion parameters and registration results, and use the TARWS data for subsequent statistical analysis.

Brain regions activated in male and female subjects during various tasks

Male tasks:

| Region Label | MNI | | | F value | Cluster Size |
| --- | --- | --- | --- | --- | --- |
|  | X | Y | Z |  |  |
| SupraMarginal_R | 69 | -24 | 27 | 10.8782 | 368 |
| Rolandic_Oper_L | -48 | 0 | 6 | 8.1561 | 158 |
| Paracentral_Lobule_R | 9 | -15 | 63 | 6.9088 | 89 |


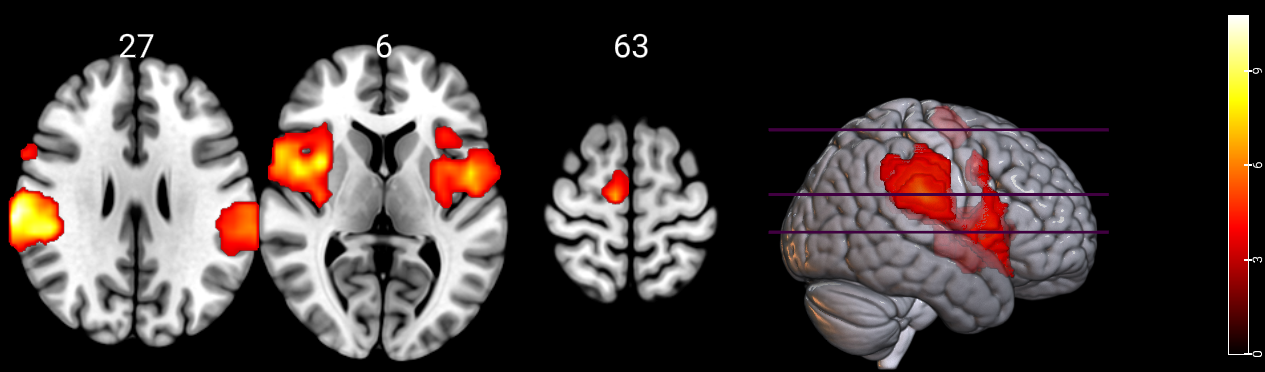


Female tasks:

| Region Label | MNI | | | F value | Cluster Size |
| --- | --- | --- | --- | --- | --- |
|  | X | Y | Z |  |  |
| Cerebelum_8_L | -18 | -78 | -51 | 6.2024 | 23 |
| SupraMarginal_R | 66 | -15 | 27 | 9.6608 | 292 |
| Postcentral_L | -54 | -18 | 18 | 8.6439 | 88 |


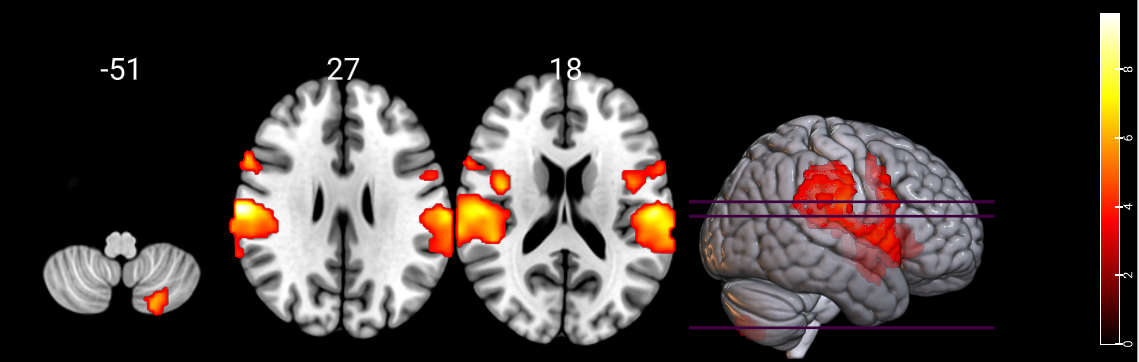


----------------------------------

Male tasks:

spmT_0001.nii,1

Type: T

df: 24

Threshold

-- p value = 0.001

-- intensity = 3.4668

-- cluster size = 90

Number of clusters found: 3

----------------------

Cluster 1

Number of voxels: 1868

Peak MNI coordinate: 69 -24 27

Peak MNI coordinate region: // undefined // undefined // undefined // undefined // undefined // SupraMarginal_R (aal)

Peak intensity: 10.8782

# voxels structure

1868 --TOTAL # VOXELS--

1749 Right Cerebrum

781 White Matter

716 Gray Matter

531 Sub-lobar

510 Parietal Lobe

499 Frontal Lobe

393 Insula

368 SupraMarginal_R (aal)

321 Rolandic_Oper_R (aal)

308 Insula_R (aal)

265 Postcentral Gyrus

245 Inferior Frontal Gyrus

224 Inferior Parietal Lobule

189 Precentral Gyrus

175 Temporal Lobe

165 Frontal_Inf_Oper_R (aal)

144 brodmann area 13

136 Postcentral_R (aal)

130 Superior Temporal Gyrus

109 brodmann area 40

100 Temporal_Sup_R (aal)

93 Precentral_R (aal)

82 Extra-Nuclear

54 brodmann area 44

47 Sub-Gyral

43 brodmann area 22

43 brodmann area 6

43 Putamen_R (aal)

38 Claustrum

37 Middle Frontal Gyrus

37 Temporal_Pole_Sup_R (aal)

33 brodmann area 45

33 brodmann area 43

33 Frontal_Inf_Tri_R (aal)

32 Frontal-Temporal Space

27 brodmann area 9

27 brodmann area 47

25 Lentiform Nucleus

23 Transverse Temporal Gyrus

23 brodmann area 3

22 brodmann area 2

21 Putamen

21 brodmann area 4

20 Frontal_Inf_Orb_R (aal)

19 brodmann area 38

18 brodmann area 42

14 Heschl_R (aal)

13 Pallidum_R (aal)

11 brodmann area 1

11 brodmann area 41

10 Parietal_Inf_R (aal)

9 Supramarginal Gyrus

7 Frontal_Mid_R (aal)

6 brodmann area 8

4 brodmann area 21

4 Lateral Globus Pallidus

----------------------

Cluster 2

Number of voxels: 1247

Peak MNI coordinate: -48 0 6

Peak MNI coordinate region: // Left Cerebrum // Frontal Lobe // Precentral Gyrus // Gray Matter // brodmann area 44 // Rolandic_Oper_L (aal)

Peak intensity: 8.1561

# voxels structure

1247 --TOTAL # VOXELS--

1187 Left Cerebrum

558 White Matter

485 Gray Matter

449 Parietal Lobe

377 Sub-lobar

277 Insula

259 SupraMarginal_L (aal)

238 Inferior Parietal Lobule

218 Insula_L (aal)

197 Postcentral Gyrus

173 Frontal Lobe

162 Temporal_Sup_L (aal)

158 Rolandic_Oper_L (aal)

152 Temporal Lobe

137 Superior Temporal Gyrus

135 brodmann area 40

108 Precentral Gyrus

92 brodmann area 13

88 Postcentral_L (aal)

87 Frontal_Inf_Oper_L (aal)

60 Extra-Nuclear

55 brodmann area 22

49 Inferior Frontal Gyrus

45 Putamen_L (aal)

36 Parietal_Inf_L (aal)

33 brodmann area 44

33 Frontal-Temporal Space

26 brodmann area 2

24 Sub-Gyral

23 Putamen

23 Lentiform Nucleus

22 Claustrum

20 brodmann area 6

20 brodmann area 42

18 brodmann area 43

14 Temporal_Pole_Sup_L (aal)

14 Heschl_L (aal)

14 Precentral_L (aal)

13 Supramarginal Gyrus

13 Frontal_Inf_Tri_L (aal)

10 brodmann area 1

10 brodmann area 45

7 brodmann area 3

5 brodmann area 4

2 Middle Temporal Gyrus

2 Transverse Temporal Gyrus

2 brodmann area 41

1 brodmann area 47

1 brodmann area 21

1 Pallidum_L (aal)

----------------------

Cluster 3

Number of voxels: 90

Peak MNI coordinate: 9 -15 63

Peak MNI coordinate region: // Right Cerebrum // Frontal Lobe // Medial Frontal Gyrus // White Matter // undefined // Supp_Motor_Area_R (aal)

Peak intensity: 6.9088

# voxels structure

90 --TOTAL # VOXELS--

90 Frontal Lobe

90 Right Cerebrum

89 Supp_Motor_Area_R (aal)

74 Medial Frontal Gyrus

54 Gray Matter

54 brodmann area 6

36 White Matter

11 Superior Frontal Gyrus

4 Middle Frontal Gyrus

1 Frontal_Sup_R (aal)

1 Precentral Gyrus

-------------------------------------------------------------------------------------------------------------------------------------------------------------------

Female tasks:

spmT_0001.nii,1

Type: T

df: 20

Threshold

-- p value = 0.001

-- intensity = 3.5518

-- cluster size = 77

Number of clusters found: 3

----------------------

Cluster 1

Number of voxels: 77

Peak MNI coordinate: -18 -78 -51

Peak MNI coordinate region: // undefined // undefined // undefined // undefined // undefined // undefined

Peak intensity: 6.2024

# voxels structure

77 --TOTAL # VOXELS--

63 Inferior Semi-Lunar Lobule

63 Left Cerebellum

63 Cerebellum Posterior Lobe

23 Cerebelum_8_L (aal)

22 Cerebelum_7b_L (aal)

6 Cerebelum_Crus2_L (aal)

----------------------

Cluster 2

Number of voxels: 1722

Peak MNI coordinate: 66 -15 27

Peak MNI coordinate region: // Right Cerebrum // Parietal Lobe // Postcentral Gyrus // undefined // undefined // SupraMarginal_R (aal)

Peak intensity: 9.6608

# voxels structure

1722 --TOTAL # VOXELS--

1676 Right Cerebrum

764 White Matter

685 Gray Matter

524 Frontal Lobe

447 Parietal Lobe

442 Sub-lobar

325 Insula

292 Rolandic_Oper_R (aal)

292 SupraMarginal_R (aal)

272 Insula_R (aal)

267 Inferior Frontal Gyrus

265 Postcentral Gyrus

226 Temporal Lobe

171 Precentral Gyrus

169 Inferior Parietal Lobule

167 Superior Temporal Gyrus

166 Frontal_Inf_Oper_R (aal)

138 Temporal_Sup_R (aal)

130 Postcentral_R (aal)

118 brodmann area 13

93 brodmann area 40

82 Precentral_R (aal)

71 Extra-Nuclear

65 Sub-Gyral

52 Middle Frontal Gyrus

50 Putamen_R (aal)

45 brodmann area 6

45 Frontal_Inf_Tri_R (aal)

42 brodmann area 44

38 brodmann area 22

37 Frontal-Temporal Space

35 brodmann area 47

33 brodmann area 9

32 Temporal_Pole_Sup_R (aal)

31 brodmann area 43

30 brodmann area 42

29 brodmann area 45

28 brodmann area 2

25 Putamen

25 Claustrum

25 Transverse Temporal Gyrus

25 Lentiform Nucleus

24 brodmann area 3

19 brodmann area 4

19 brodmann area 38

18 Frontal_Mid_R (aal)

18 brodmann area 41

17 Frontal_Inf_Orb_R (aal)

13 brodmann area 1

9 Heschl_R (aal)

8 brodmann area 8

5 brodmann area 21

4 Supramarginal Gyrus

3 Parietal_Inf_R (aal)

1 Pallidum_R (aal)

----------------------

Cluster 3

Number of voxels: 1468

Peak MNI coordinate: -54 -18 18

Peak MNI coordinate region: // Left Cerebrum // Parietal Lobe // Postcentral Gyrus // White Matter // undefined // Postcentral_L (aal)

Peak intensity: 8.6439

# voxels structure

1468 --TOTAL # VOXELS--

1457 Left Cerebrum

669 White Matter

617 Gray Matter

462 Sub-lobar

359 Parietal Lobe

329 Insula

299 Frontal Lobe

297 Temporal Lobe

281 Insula_L (aal)

262 Temporal_Sup_L (aal)

230 Superior Temporal Gyrus

198 Postcentral Gyrus

192 SupraMarginal_L (aal)

171 Rolandic_Oper_L (aal)

158 Inferior Parietal Lobule

139 Inferior Frontal Gyrus

130 Frontal_Inf_Oper_L (aal)

122 Precentral Gyrus

106 brodmann area 13

92 brodmann area 40

88 Postcentral_L (aal)

70 brodmann area 22

69 Precentral_L (aal)

68 Extra-Nuclear

54 Putamen_L (aal)

51 Sub-Gyral

44 Putamen

44 Lentiform Nucleus

43 Temporal_Pole_Sup_L (aal)

42 brodmann area 42

40 Frontal-Temporal Space

40 Transverse Temporal Gyrus

39 brodmann area 44

35 brodmann area 6

26 Frontal_Inf_Orb_L (aal)

26 brodmann area 41

25 Claustrum

24 brodmann area 2

22 Frontal_Inf_Tri_L (aal)

20 brodmann area 43

19 Parietal_Inf_L (aal)

15 brodmann area 9

14 brodmann area 47

13 Heschl_L (aal)

13 brodmann area 45

10 brodmann area 1

10 brodmann area 38

10 brodmann area 21

9 brodmann area 3

7 Middle Frontal Gyrus

5 brodmann area 4

3 Supramarginal Gyrus

3 Pallidum_L (aal)

1 Middle Temporal Gyrus

1 brodmann area 8
