# Supplementary material for: Gender differences in brain activity underlying acupuncture sensations at LR3: a task-based fMRI study
Source: Front Hum Neurosci. 2025 Sep 9;19:1649644. doi: 10.3389/fnhum.2025.1649644 (PMC12454379; doi:10.3389/fnhum.2025.1649644)

**Functional connectivity analysis process and results**

**Data preprocessing**

T Delete the first 10 time points because the scan has just started and the signal has not yet reached a stable state. The subjects also need some time to adapt to the scan noise. According to the experimental design, delete the first 20 seconds of the resting state.

A slice timing time layer correction (36 layers, layer sequence [2:2:36 1:2:35], reference 36)

When the MRI machine scans, it acquires images of the brain layer by layer, rather than obtaining a complete image of the brain instantaneously. However, post-processing software assumes that the 3D brain is acquired simultaneously, so time layer correction is required to use interpolation to make all layers appear as if they were acquired simultaneously.

R realign Head motion correction

Using the average functional image as a reference, adjust the position of the brain at each time point to ensure that the data direction remains consistent across all time points, thereby reducing noise caused by head motion during scanning. Simultaneously record the extent of head motion for each subject, using this as a standard to assess data quality and exclude subjects with excessive head motion.

W normalize Spatial normalization Select the DARTEL method for registration. This method creates a group-level structural image template based on the T1 structural images of all current subjects, enabling registration of all subjects from individual space to standard MNI space, thereby effectively achieving spatial normalization of individual brains.

Based on these preprocessing steps, we further employed linear regression to remove irrelevant noise time series (including head motion-induced Friston-24, cerebrospinal fluid, and white matter signals, as well as their time-differenced signals (derivatives)) and task-related signals. This processing was fully conducted in accordance with Cole, M. W., Ito, T., Schultz, D., Mill, R., Chen, R., & Cocuzza, C. (2019). Task activations produce spurious but systematic inflation of task functional connectivity estimates. Neuroimage, 189, 1-18. This method employs the FIR task regression approach, which has been widely applied in task-based functional connectivity analysis. Specifically, the FIR regression method involves fitting the average response at each time point within a fixed window length, which is locked to the experimental block corresponding to a given task condition.In the fitting process, the window length for each condition is matched to the duration of the event, plus an additional 16 seconds (8 regression variables) to account for the potential duration of hemodynamic responses. From a practical computational perspective, FIR regression is nearly equivalent to subtracting the average evoked response, which is the standard method used in spike-related literature to eliminate task-induced activations that cause “inflation” in functional connectivity calculations. This is particularly useful for fMRI data with delayed hemodynamic responses.After the regression step, the detrend function is applied to remove the linear trend from the time series signal. Finally, considering that task-related signals may be present at high frequencies, we applied a bandpass filter with a bandwidth of 0.2 Hz to 0.008 Hz.

**Whole-brain functional connectivity based on seed points**


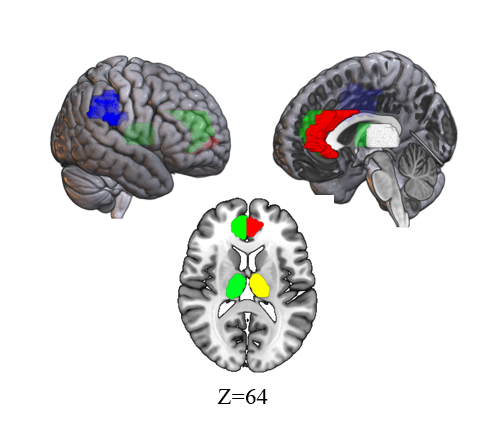


Seed points selected based on AAL

Seed points were selected based on the AAL atlas (https://www.gin.cnrs.fr/en/tools/aal/). The left thalamus, left and right anterior cingulate cortex, paracingulate cortex, and right superior temporal gyrus were extracted as templates. For each subject, the average time-series signals of each brain region were extracted. Then, the Pearson correlation algorithm was used to calculate the functional connectivity values from each brain region to every voxel in the whole brain.The correlation values R were transformed into Z values using Fisher’s Z transformation for subsequent statistical analysis. After obtaining the whole-brain functional connectivity maps for each participant based on the five seed points, we calculated the group-average functional connectivity maps for each group of participants using a one-sample t-test and compared the intergroup differences in whole-brain functional connectivity at each seed point between male Task 2 and female Task 1 using a two-sample t-test.Since there were five seed points, significant differences were adjusted to p < 0.01 based on strict multiple comparison correction. For each brain map, we applied a cluster-level multiple comparison correction method, where cluster p < 0.01 and voxel p < 0.001 were considered to indicate significant intergroup differences.

The results are as follows:

| **前扣带和旁扣带脑回** | **31** | **Cingulum_Ant_L** |  |
| --- | --- | --- | --- |
| **前扣带和旁扣带脑回** | 32 | Cingulum_Ant_R |  |
| **缘上回** | 64 | SupraMarginal_R |  |
| **丘脑** | 77 | Thalamus_L |  |
| **丘脑** | 78 | Thalamus_R |  |

Differences between the two groups in the right lateral margin of the seed point of the whole brain FC:

|  |  |  | MNI | | |
| --- | --- | --- | --- | --- | --- |
| Region Label | Extent | t-value | x | y | z |
|  |  |  |  |  |  |
| Cerebellum_6_L | 36 | 5.500 | -24 | -60 | -30 |
| Cingulate_Mid_R | 44 | 4.882 | 9 | 6 | 33 |
| Putamen R | 41 | 4.864 | 39 | -12 | -9 |


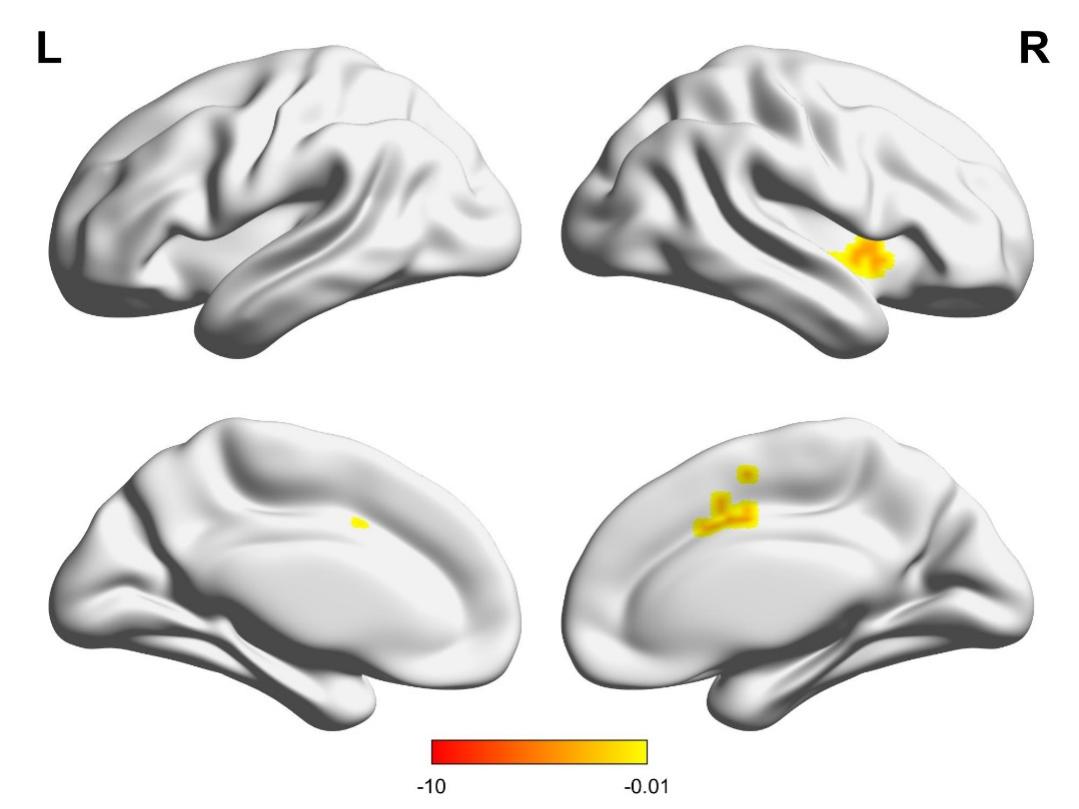


Differences between the two groups in whole-brain FC of the left thalamic seed point：

|  |  |  | MNI | | |
| --- | --- | --- | --- | --- | --- |
| Region Label | Extent | t-value | x | y | z |
|  |  |  |  |  |  |
| Caudate_L | 176 | 7.383 | -18 | 9 | 15 |
| Caudate_R | 176 | 5.739 | 12 | 3 | 12 |
| Precuneus_R | 176 | 5.287 | 9 | -39 | 3 |
| Calcarine_L | 197 | 6.592 | -3 | -57 | 3 |
| Lingual_R | 197 | 5.565 | 6 | -78 | -6 |
| Lingual_L | 197 | 4.523 | -12 | -39 | -3 |
| Postcentral_L | 44 | 5.482 | -30 | -30 | 54 |
| Supp_Motor_Area_L | 47 | 5.135 | 0 | 12 | 45 |


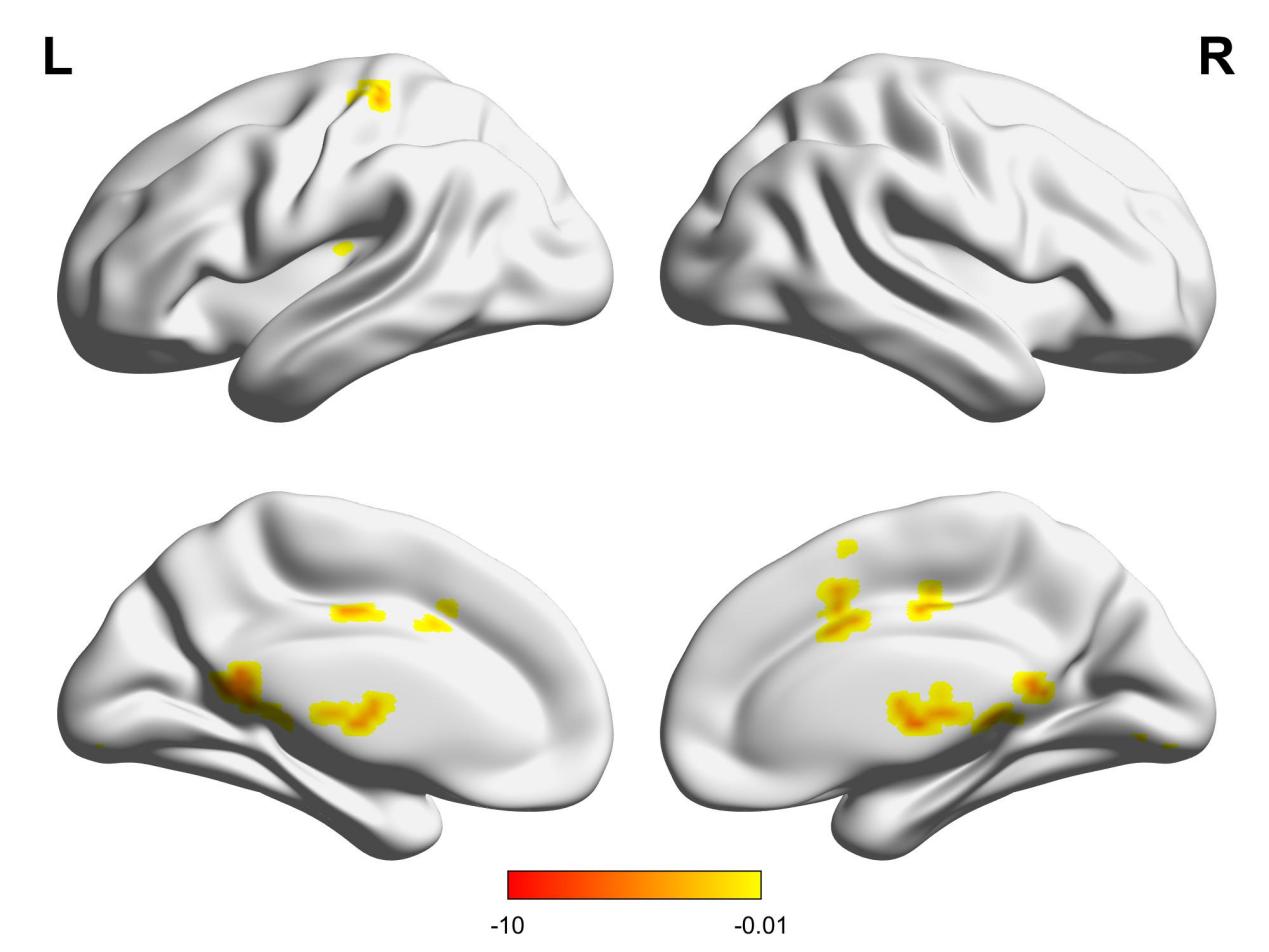


Differences between the two groups in whole-brain FC of the right thalamic seed point:

|  |  |  | MNI | | |
| --- | --- | --- | --- | --- | --- |
| Region Label | Extent | t-value | x | y | z |
|  |  |  |  |  |  |
| Calcarine_L | 118 | 9.999 | -3 | -57 | 3 |
| Cingulate_Post_R | 118 | 4.803 | 9 | -42 | 21 |
| Lingual_R | 123 | 7.194 | 9 | -42 | 3 |
| Precuneus_L | 342 | 6.735 | -9 | -48 | 45 |
| Paracentral_Lobule_L | 342 | 6.093 | -6 | -21 | 54 |


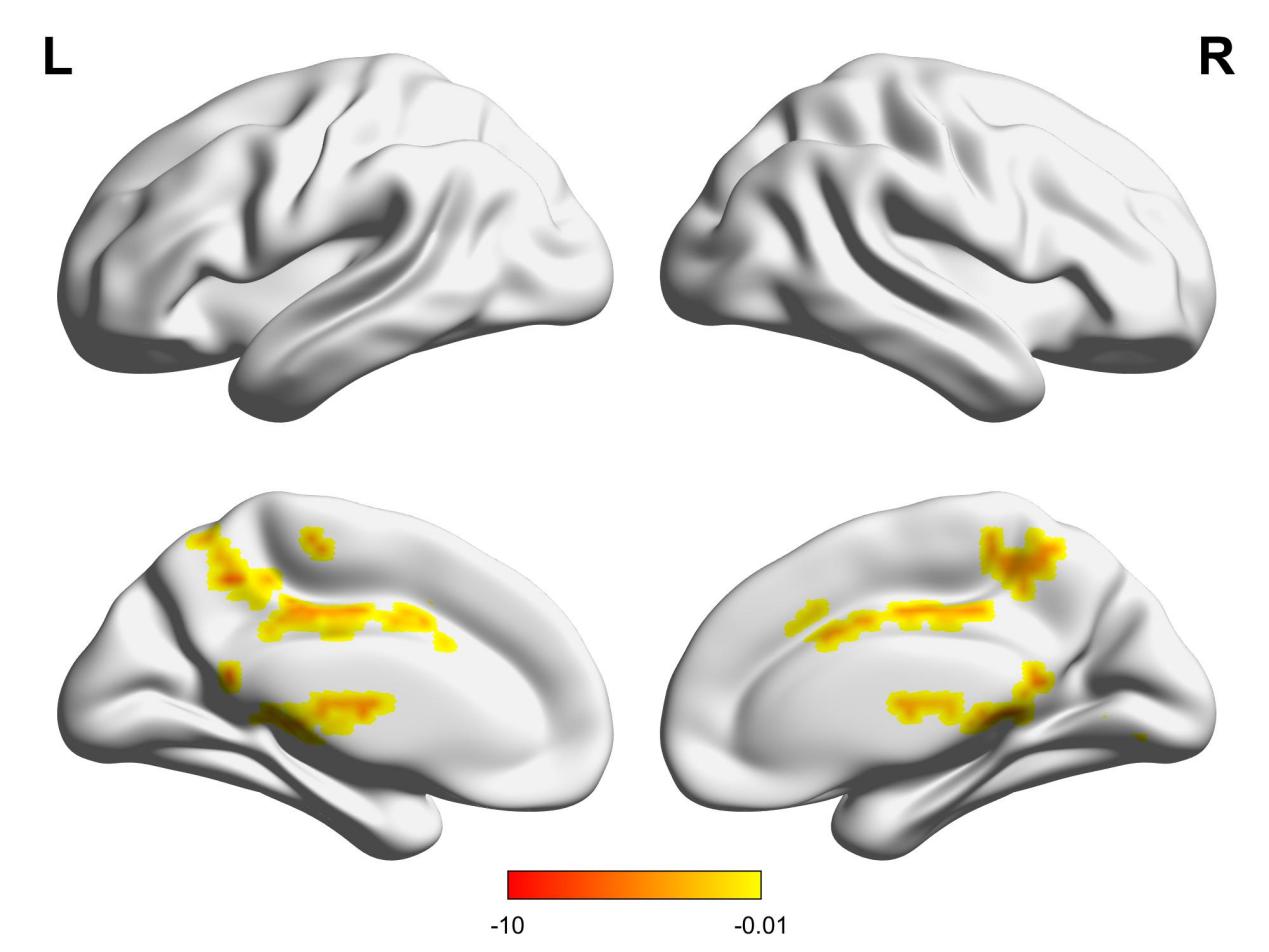

Supplement: Supplementary file 1 [file Supplementary_file_1.zip › Supplementary Material/Table_2.DOCX]
